# Clinical Characteristics and Prognostic Risk Factors of Parasellar Chondrosarcoma

**DOI:** 10.3390/brainsci12101353

**Published:** 2022-10-06

**Authors:** Linpeng Zhang, Chen Wang, Xueling Qi, Xufei Zhang, Changxiang Yan, Ning Liu, Yakun Yang, Ming Ren, Yabo Liu, Xiaojun Fu, Song Han, Xianwei Zeng

**Affiliations:** 1 Department of Neurosurgery, Qilu Hospital of Shandong University, Jinan 250012, China; 2 School of Medicine, Shandong University, Jinan 250012, China; 3 Department of Neurosurgery, Sanbo Brain Hospital, Capital Medical University, Beijing 100093, China; 4 Department of Imaging, Sanbo Brain Hospital, Capital Medical University, Beijing 100093, China; 5 Department of Neurology, Sanbo Brain Hospital, Capital Medical University, Beijing 100093, China

**Keywords:** parasellar chondrosarcoma, clinical features, imaging features, pathology, prognosis

## Abstract

Background: Parasellar chondrosarcomas are extremely rare. This study describes the characteristics of parasellar chondrosarcoma and analyzes the risk factors and prognosis based on the resection degree. Methods: Fifteen patients with pathologically diagnosed parasellar chondrosarcoma were retrospectively analyzed for the clinical data, surgical methods, and prognosis to identify relationships between the surgical resection degree, tumor recurrence, and imaging characteristics. Results: Twelve patients had eye dysfunction and ptosis. Differentiation from other parasellar tumors by imaging is difficult. The preoperative Karnofsky Performance Scale (KPS) score positively correlated with the tumor resection degree (*p* = 0.026) and negatively correlated with the maximum tumor diameter (*p* = 0.001). Tumor recurrence negatively correlated with the resection degree (*p* = 0.009). The postoperative KPS score positively correlated with the preoperative KPS score (*p* < 0.001) and tumor resection degree (*p* = 0.026), and negatively correlated with the maximum tumor diameter (*p* = 0.016) and age (*p* = 0.047). An improved KPS score positively correlated with the tumor resection degree (*p* = 0.039). Patients who underwent total resection of the chondrosarcoma had longer progression-free survival than those who underwent partial resection (*p* = 0.0322). Conclusion: Parasellar chondrosarcomas are difficult to resect completely. Preoperative KPS score is an important factor for the degree of resection. KPS score, age, maximum tumor diameter, and resection degree may be important prognostic factors.

## 1. Introduction

Chondrosarcoma of the skull base is a rare brain tumor with low metastatic potential [1], accounting for only 1.5% of all intracranial tumors [2]. It is considered to be cartilage residue caused by the incomplete ossification of the cartilaginous skull [3]. Because of the special anatomical location, chondrosarcoma of the skull base often invades the petroclival area, parasellar area, and jugular foramen area; causing the erosion of important arteries and cranial nerves [4,5,6,7,8]; and penetrating the dura mater to compress the brain stem [8]. Therefore, achieving complete removal by surgery is very challenging [8,9].

Only 10% of the sellar and parasellar tumors originate from the non-pituitary region [10], which is an anatomically complex region bordering the sella turcica. Their impact on important anatomical structures can cause extensive clinical manifestations [11]. Parasellar chondrosarcoma is extremely rare, and patients often complain of dizziness and diplopia due to the compression of cranial nerves and intracranial vessels [4,9,12,13,14,15,16,17,18,19,20,21]. Volpe et al. found that ocular symptoms such as diplopia or visual impairment are the initial symptoms of skull base chondrosarcoma and chordoma, and the incidence of these symptoms in these two diseases is very similar [22]. Chordoma originates from the residues of the notochord and can also invade the parasellar region [23], cranial base chondrosarcoma and chordoma usually show low signal intensity on T1-weighted magnetic resonance imaging (MRI) and high signal intensity on T2-weighted MRI, while there is no obvious imaging difference on contrast-enhanced T1-weighted MRI [24]. As such, chordoma is frequently misdiagnosed as chordoma because of the molecular and imaging similarities [25]. As with chondrosarcoma of the skull base, parasellar chondrosarcoma has four different types: conventional, mesenchymal, clear cell, and differentiated [2]. The World Health Organization (WHO) classifies these tumors as grade 1 (high differentiation), grade 2 (medium differentiation), or grade 3 (low differentiation) [26]. The pathological results are very important when differentiating between parasellar chondrosarcoma and chordoma [27]. For example, the analysis of the expression of the Brachyury gene shows that a transcription factor is highly expressed in chordoma [28,29], while chondrosarcoma does not respond to keratin and epileptelial membrane antigen, S-100. In addition, epileptelial membrane antigens (EMA) are positive [24], which are often used to distinguish between parasellar chondrosarcoma and chordoma.

The invasiveness of skull base chondrosarcoma greatly increases the difficulty of surgical resection. Based on maximum tumor resection, adjuvant radiotherapy is often used to control residual tumors [7]. Although proton therapy protects key parts at a higher therapeutic dose [7], patients with parasellar chondrosarcoma are at a higher risk of vision loss after treatment [25]. In addition, because of the special location of the parasellar chondrosarcoma, the tumor often wraps around the internal carotid artery and invades the nerves passing through the cavernous sinus [4,5,6,7,8]. Therefore, the surgical strategy for parasellar chondrosarcoma should involve removing the tumor to alleviate the space-occupying effect and protect the nerves and blood vessels during the operation.

Information regarding parasellar chondrosarcoma is sparse. Therefore, we described 15 cases of parasellar chondrosarcoma and analyzed the risk factors and prognosis based on the resection degree.

## 2. Materials and Methods

### 2.1. Patient Population

**Figure 1:**
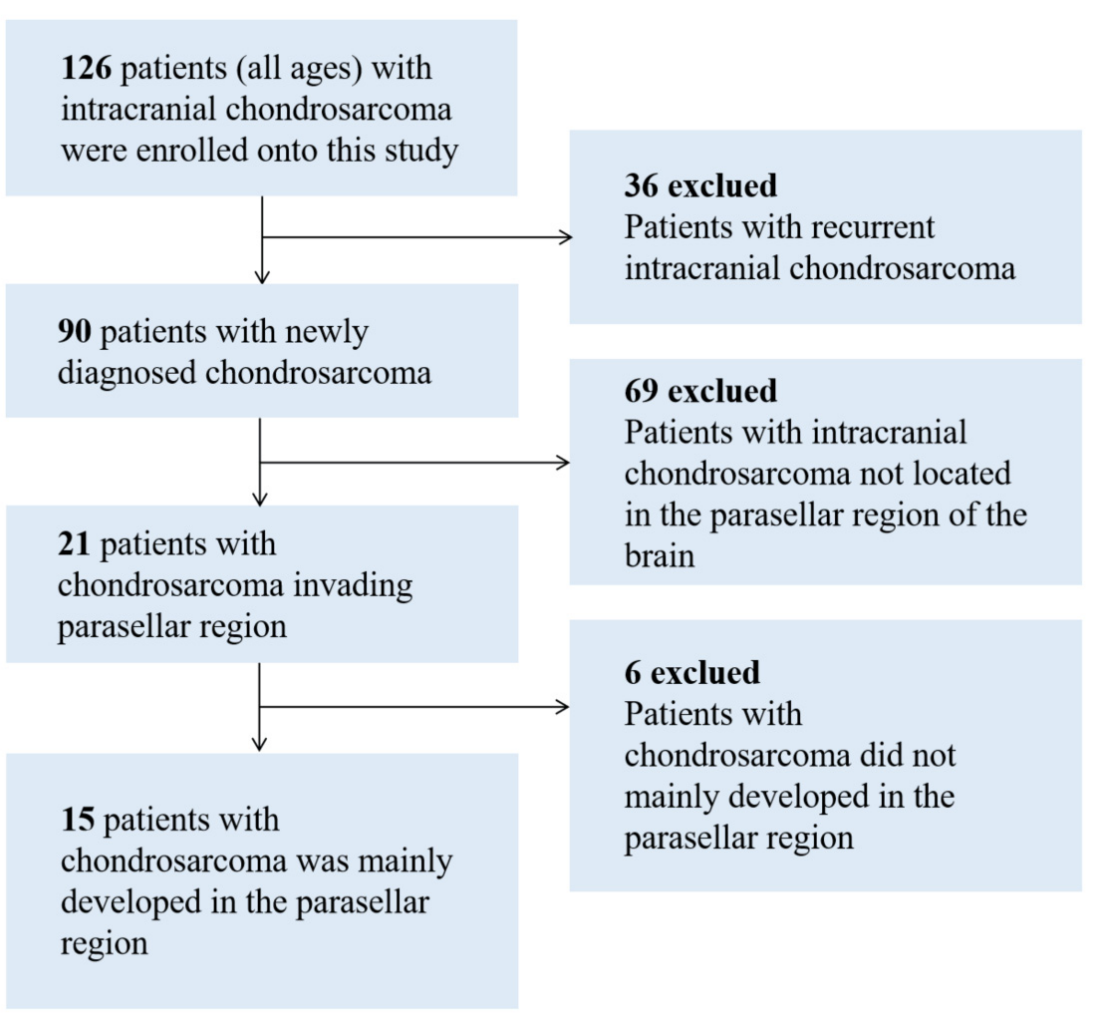
Flow chart of inclusion patients.

Inpatients diagnosed with parasellar chondrosarcoma at the Sanbo Brain Hospital of Capital Medical University, Beijing, China between 2013 and 2022 were selected. The inclusion criteria were: (1) first-onset patients; (2) imaging-confirmed lesions located in parasellar region; and (3) histopathology-confirmed parasellar chondrosarcoma. Those with chondrosarcoma located in the intracranial position outside the parasellar region, recurrent chondrosarcoma, metastatic brain chondrosarcoma, or other brain tumors or diseases were excluded. Finally, 15 patients with parasellar chondrosarcoma were included in the analyses (Figure 1). Patients were divided into children’s cases (≤14 years old), adolescent and young adult cases (14–40 years old), adult cases (40–65 years old), and elderly cases (>65 years) [30]. Patients who died were followed up to the date of death, and those who survived were followed up to 2 June 2022.

### 2.2. Radiological Profiles

All patients underwent preoperative MRI, computed tomography (CT), and CT angiography (CTA) (Table 1). Preoperative MRI sequences included T1 and enhanced T1-weighted, T2 and enhanced T2-weighted, and fluid-attenuated inversion recovery (FLAIR) and enhanced FLAIR imaging. The tumor location and size were evaluated by MRI, while bone destruction, osteogenesis, and calcification were assessed by CT. In addition, the relationships between the tumor and important cerebral vessels were evaluated by CTA. All radiologic data (pre- and postoperative) were retrospectively examined by a neuroradiologist who was blinded to the histological examination results, surgical procedures, and clinical conditions; the tumor size, tumor location, relationship with large vessels, resection degree, and recurrence were assessed. Total resection was defined as 100% removal of the tumor, near-total resection as ≥90% removal (excluding 100%), and partial resection as <90% removal based on the intraoperative findings and postoperative MRIs [31]. The volume of the tumor was measured by “miplatform ZFP viewer” software.

### 2.3. Histopathological Examinations

Histopathological examinations were performed according to the WHO classification of chondrosarcoma [26]: central atypical chondrosarcoma, secondary peripheral atypical chondrosarcoma, central chondrosarcoma, secondary peripheral chondrosarcoma, periosteal chondrosarcoma, clear cell chondrosarcoma, mesenchymal chondrosarcoma, and dedifferentiated chondrosarcoma.

#### 2.3.1. Central Atypical Cartilaginous Tumor/Chondrosarcoma, Grade 1 (ACT/CS1)

Yellowish-white chalky areas of calcification are often present in tissue fragments; in specimens, the tumor is usually sharply demarcated and may demonstrate the erosion of the surrounding cortex; soft tissue extension is usually not seen in central ACT/CS1 [26].

The cellularity in central ACT/CS1 is low; the nuclei can be small and condensed (lymphocyte-like) and generally uniform in size; binucleation is frequently seen, while mitoses are absent. The surrounding matrix is predominantly hyaline, sometimes with more mucoid or myxoid changes [26]. Necrosis may be present. Areas of a pre-existing enchondroma with extensive calcifications may be recognized in central ACT/CS1 [26].

#### 2.3.2. Central Chondrosarcoma, Grade 2 and 3

Cystic changes can be seen in resection specimens, as well as areas of myxoid material. Calcification can be visible as yellowish-white chalky areas. Erosion and destruction of the cortex with soft tissue extension are frequently present [26].

The cellularity is higher than in ACT/CS1, and mitoses are present, the nuclei can still be small and condensed; more often, the nuclei vary in size, with open chromatin and a visible nucleolus; nuclear atypia can be present but is usually not severe; binucleation can be seen, and necrosis can be present [26]. In grade 3 chondrosarcomas, mitoses are more easily found, and the cells at the periphery of the mostly myxoid, highly cellular tumor lobules are spindled and less differentiated [26].

#### 2.3.3. Immunohistochemistry

Paraffin sections were dewaxed, and gradient alcohol was added to water. Distilled water was then rinsed for 2 min × 3 times, after which slices were placed into the pressure cooker with antigen repair solution, heated in the electromagnetic furnace, and counted 2 min and 30 s after the safety valve was injected. Samples were cooled at room temperature after the safety valve was deflated and rinsed in PBS (Beijing Zhongshan Jinqiao, Beijing, China) for 2 min × 3 times. Then, sections were stained with normal goat serum blocked for 2 h and then incubated with the following primary antibodies: anti-S100 beta antibody [EP1576Y]—Astrocyte Marker (1:200, ab52642, Abcam, Cambridge, UK); D2-40 (1:100, Beijing Zhongshan Jinqiao, Beijing, China); anti-Brachyury/Bry antibody [EPR18113] (1:8000, ab209665, Abcam, Cambridge, UK); CK (1:100, Beijing Zhongshan Jinqiao, Beijing, China); anti-p53 antibody [E26] (1:100, ab32389, Abcam, Cambridge, UK); anti-Ki67 antibody [SP6] (1:200, ab16667, Abcam, Cambridge, UK); IDH1 r132h (1:50 dianova, Hamburg, Germany) at 4 °C overnight. Consequently, slices were rinsed PBS for 2 min × 3 times and incubated with an appropriate proportion of diluted secondary antibody (anti rabbit IgG H & L, Abcam, Cambridge, MA, USA) at 37 °C for 20 min. Finally, samples were rinsed with PBS 2 min × 3 times; the nuclei were counterstained with hematoxylin for 1 min and then trod with 1% hydrochloric acid alcohol for several seconds, exposed to gradient alcohol dehydration, sealed with neutral gum, and observed under a microscope (Leica dm3000, Mannheim, Germany).

All patients with parasellar chondrosarcoma were treated with a routine surgical resection to obtain pathological tissues for histological examination. All pathological data were retrospectively examined by a neurologist who was blinded to the surgical procedure, imaging data, and clinical conditions.

### 2.4. Karnofsky Performance Scale

The preoperative Karnofsky Performance Scale (KPS) score can roughly evaluate the quality of life of tumor patients before and after surgery [32]. The higher the score, the better the quality of life and prognosis. 

### 2.5. Statistical Analyses

SPSS 22.0 software (SPSS Inc., Chicago, IL, USA) was used for the statistical analysis. Kaplan–Meier curves were used to calculate overall survival (OS) and progression-free survival (PFS). The K-S test was used for the normality test, and a Spearman rank correlation analysis was used for the correlation analysis. A *p*-value < 0.05 was considered to be statistically significant.

## 3. Results

### 3.1. Patient Demographics

Among the 15 patients who were included in the study, 12 patients were WHO grade 1 and 3 were grade 2. The median age was 34 years (range 14–69), and the male-to-female ratio was 7:8 (Table 1). There were one child case (≤14 years old), 8 adolescent and young adult cases (14–40 years old), 5 adult cases (40–65 years old), and 1 elderly patient (>65 years) included (Table 1). The primary clinical manifestations of hospitalized patients were eye function and movement disorders (12 patients, 80%), dizziness (3 patients, 20%), and encephalagia (8 patients, 53.33%; Table 1).

### 3.2. Radiological Profiles

**Figure 2:**
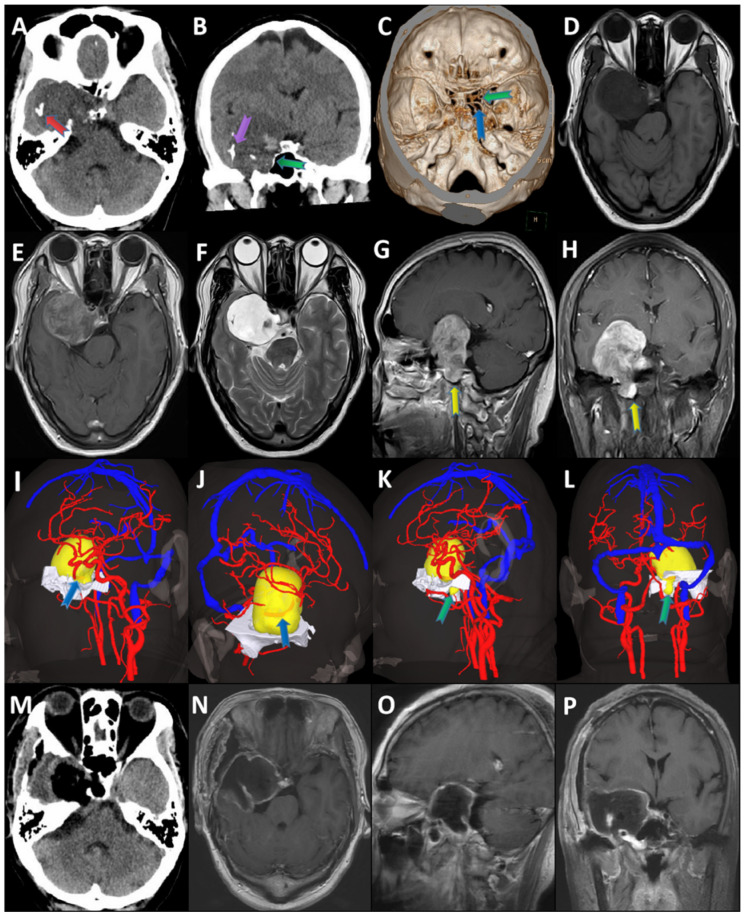
The preoperative, intraoperative, and postoperative images of right parasellar chondrosarcoma were displayed. (**A**): Preoperative CT scan showed mixed density shadow near the right parasellar, the position indicated by the red arrow in (**A**) is the bone composition in the tumor; (**B**,**C**): preoperative CT bone window (**B**) and CTA imaging reconstruction (**C**) showed that the bone destruction (indicated by the green arrow) and osteogenesis (indicated by the purple arrow) can be seen before operation, while important arteries were compressed by the tumor (indicated by the blue arrow); (**D**–**H**): the main body of tumor showed long T1 on T1-weighted image MRI before operation in (**D**), the position indicated by the yellow arrow in (**G**,**H**) showed tumor surrounded extracranial, preoperative T2-weighted image MRI showed that the tumor was long T2 in (**E**,**G**,**H**,**F**): MRI enhancement showed that the tumor was significantly enhanced; (**I**–**L**): MRI 3D reconstruction, the position indicated by the blue arrow in (**I**,**J**) showed internal carotid artery surrounded by tumor, the position indicated by the green arrow in K and L showed tumor surrounded extracranial; (**M**): after operation, CT plain scan showed low density shadow near the right parasellar, which was normal after operation; (**N**–**P**): right parasellar tumor was excised on T1 and T2 MRI after operation.

All 15 chondrosarcoma cases were located in the parasellar (Table 1). The maximum diameter was 40 ± 13 mm. The CT examinations showed a slightly high or mixed density shadow and a patchy calcification shadow in the tumor. Furthermore, the CT examination identified bone destruction in seven cases (Figure 2B,C,K,L and Table 1) and two cases of osteogenesis (Figure 2A). A T1-weighted brain MRI showed a mixed signal dominated by low signal intensity (Figure 2D and Supplementary Table S1), while the T2-weighted MRI showed a mixed signal dominated by high signal intensity (Figure 2E,G,H and Supplementary Table S1), and these imaging characteristics were similar to those of chondrosarcoma located in other parts of the body. A contrast-enhanced MRI revealed the obvious enhancement of the tumor (Figure 2F), and CTA and imaging reconstruction demonstrated that the tumor surrounded important large arteries (Figure 2C,I,J and Table 1). For example, the tumor surrounded the internal carotid artery in four cases; the middle cerebral artery in one case; the internal carotid and middle cerebral arteries in one case (Figure 2C); the internal carotid and posterior cerebral arteries in one case; the internal carotid, middle cerebral, and posterior cerebral arteries in two cases; and the internal carotid, vertebral, and basilar arteries in one case. However, in five cases, the tumor did not surround any large vessels. After the operation, CT images showed a low density shadow in the tumor area (Figure 2M), and the tumor sites in the T1 and T2-weighted MRI results were excised (Figure 2N,O,P).

### 3.3. Surgical Resection

**Figure 3:**
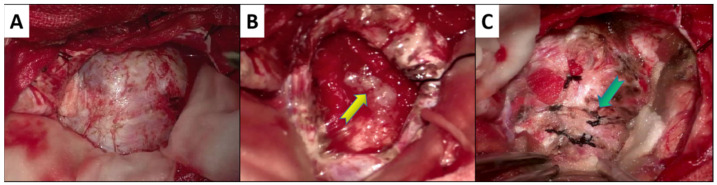
Surgical resection of parasellar chondrosarcoma. (**A**) Tumor exposure; (**B**) intraoperative condition; (**C**) total tumor resection. After the tumor was exposed, it was found that the tumor was located in the cavernous sinus (**A**), and local calcification could be seen inside the tumor. The yellow arrow shows the calcified part (**B**). After the tumor was removed, the green arrow shows that the gel sponge was filled into the cavernous sinus for full hemostasis, and the lateral wall of the cavernous sinus was sutured to prevent the gel sponge from falling into the cavernous sinus due to the cerebrospinal fluid been surrounded (**C**).

Fifteen patients with chondrosarcoma underwent surgical resection. In three cases (near-total resection), an endoscopic transsphenoidal approach was used. A frontotemporal craniotomy was performed in 10 cases, of which 6 cases were treated with total resection, 1 case was treated with near total resection, and 3 cases were treated with partial resection. The frontotemporal zygomatic arch approach was used in two cases; partial resection was performed in one case, while near total resection was performed in another. Six cases underwent total resection (Figure 2M–P and Figure 3A,B), which was not an option for five near-total resection cases and four partial resection cases as the tumor was close to important structures (Table 2). 

The tumor resection degree was positively correlated with the preoperative KPS score (*p* = 0.026), but not with important vessel wrapping (*p* = 0.705) or the maximum tumor diameter (*p* = 0.803; Table 3). However, the preoperative KPS score negatively correlated with the maximum tumor diameter (*p* = 0.001).

### 3.4. Histopathology

**Figure 4:**
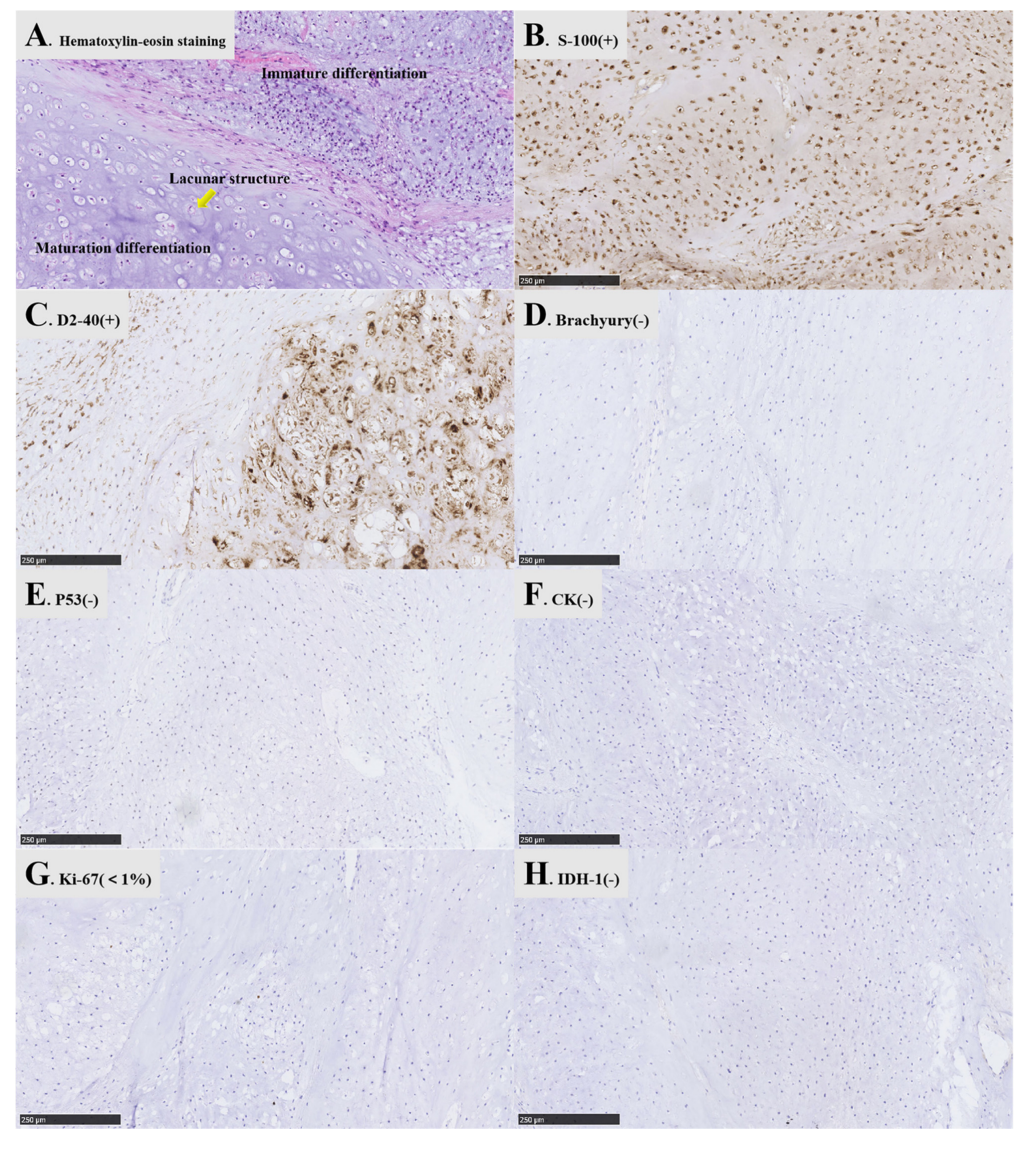
Pathological results at 10x magnification of parasellar chondrosarcoma. (**A**): The tumor is lobulated, partially differentiated, and mature, partially immature, and lacuna structure can be seen in the mature area; (**B**): S-100 positive, the cytoplasm and nucleus were positive in immunohistochemistry; (**C**): D2-40 positive, a specific indicator of chondrosarcoma; (**D**): Brachyury negative, an important molecular marker for differentiating chondrosarcoma and chordoma. Brachyury is positive in chondrosarcoma and negative in chordoma; (**E**): P53 negative, a tumor suppressor gene; (**F**): CK negative, it is positive in chordoma and negative in chondrosarcoma, which is an important molecular indicator to distinguish chondrosarcoma from chordoma; (**G**): Ki-67 low expression, the proliferation of representative cells is inactive; (**H**): IDH-1 negative.

Macroscopically, most tumors were grayish-white, hard, and tough. Furthermore, they had a general blood supply, a honeycomb-like appearance, and had clear boundaries (Figure 3A–C). A few tumors were grayish-red and slightly soft. Microscopically, at 10× magnification, the tumor (WHO grade 1) was lobulated, partially differentiated, and mature, partially immature, and a lacuna structure could be seen in the mature area (Figure 4A). Pathologically, nine cases were isocitrate dehydrogenase 1 (IDH1)-negative (Figure 4H), and six did not detect IDH1 mutation. However, all cases were S100 (Figure 4B) and D2-40 (Figure 4C) positive, and brachyury (Figure 4D), P53 (Figure 4E), and CK (Figure 4F) negative, with low Ki-67 expression (<5%) (Figure 4G).

### 3.5. Prognosis and Adjuvant Therapy

No deaths occurred during the follow-up period (December 2013 to June 2022), and all patients had a better postoperative score compared to the preoperative KPS score (Table 2). In addition, only two patients relapsed 4–8 years after the operation.

Multivariable correlation analysis results are shown in Table 4. Tumor relapse was negatively correlated with the tumor resection degree (*p* = 0.009) but did not correlate with the postoperative KPS score (*p* = 0.173), maximum tumor diameter (*p* = 0.322), WHO grade (*p* = 0.484), important vascular surrounded by tumor (*p* = 0.622), and age (*p* = 0.628). Furthermore, the postoperative KPS score positively correlated with the preoperative KPS score and tumor resection degree (*p* < 0.001 and *p* = 0.026, respectively) and negatively correlated with the maximum tumor diameter and age (*p* = 0.016 and *p* = 0.047, respectively). The KPS growth value was obtained by subtracting the preoperative KPS score from the postoperative KPS score, thus eliminating individual differences. A correlation analysis was between specific growth value and tumor resection degree. KPS growth was positively correlated with the tumor resection degree (*p* = 0.039), but not with the maximum tumor diameter (*p* = 0.194), WHO grade (*p* = 0.496), age (*p* = 0.771), and important vascular surrounded by tumor (*p* = 0.949).

**Figure 5:**
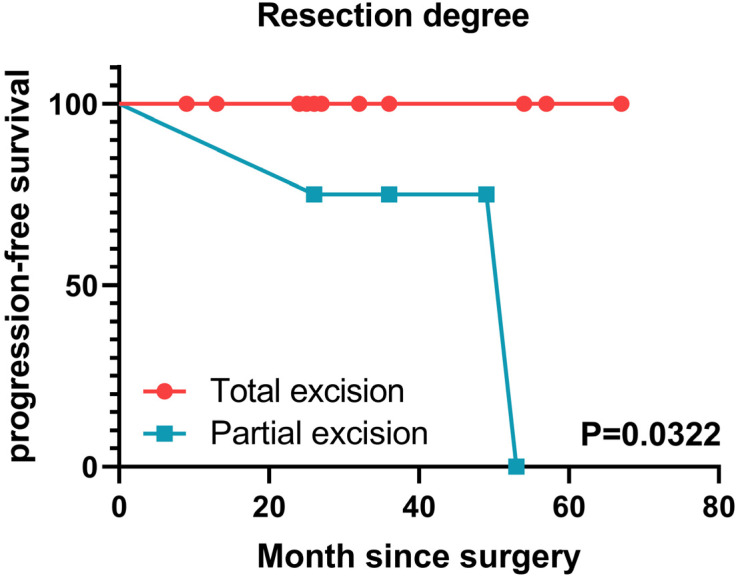
Kaplan–Meier survival analysis of 15 patients, the score on the y-axis refers to the non-recurrence rate of the patient. Patients with total resection of chondrosarcoma had longer progression-free survival than patients with partial resection (*p* = 0.0322).

The Kaplan–Meier survival analysis demonstrated that patients with total chondrosarcoma resection had longer progression-free survival than patients with partial resection (*p* = 0.0322; Figure 5). Three patients received 15 rounds of radiotherapy six months after the operation every other day for one month. Two patients had residual tumors, and one had a malignant tendency because the tumor was WHO grade 2, so radiotherapy was performed. Among them, the radiation treatment volume of patients 11 and 15 were 6800 rads, and the radiation treatment volume of patient 12 was 6600 rads. One patient made his own decision and received 4 gamma knife treatments due to his radical treatment viewpoint, and one patient was about to receive cellular immunotherapy. The remaining patients did not require adjuvant therapies (e.g., radiotherapy or chemotherapy; Table 3). However, some postoperative complications were observed, including tumor recurrence (13.33%), cerebral infarction (20%), epidural effusion (13.33%), mastoiditis (26.67%), epidural hemorrhage (13.33%), subarachnoid hemorrhage (6.67%), and arachnoid cysts (6.67%).

## 4. Discussion

Chondrosarcoma is a slow-growing and extremely rare intracranial tumor. Due to its unique anatomical structure, the parasellar tumor easily invades the sellar and cavernous sinuses, large vessels, and cavernous sinus nerves [4,5,6,7,8]. Thus, its treatment is challenging [33]. Due to the high similarity in morphology and imaging between chondrosarcoma and chordoma in the midline region of the skull base, diagnosis is also difficult [34]. It has been reported that 37% of the cases (74 of 200 cases) were misdiagnosed with chordoma before operation and pathologically confirmed as chondrosarcoma after operation [34]. Chordomas arise from the remnants of notochord, which can also be present in the parasellar region, but they are midline and usually lack calcification [34]. Parasellar chondrosarcoma and chordoma often require further molecular and genetic evaluation for a definite diagnosis [35]. Both chordoma and chondrosarcoma may be positive for S-100 and vimentin [24,26]. However, chordomas are usually immunopositive for epithelial markers such as cytokeratin and endothelial membrane antigen (EMA), whereas chondrosarcoma is negative for both markers [24]. “Brachyury” is a new, recently discovered specific diagnostic marker for chordoma, and a high expression of “Brachyury” was found in chordoma tissue samples [9,29,36].

This study comprehensively analyzed the clinical data, treatment, and prognosis of 15 patients with WHO grade 1 and 2 parasellar chondrosarcomas and evaluated the risk factors related to the resection degree and tumor recurrence. We found that most patients with parasellar chondrosarcoma were middle-aged, and the incidence rate in men and women was similar. Furthermore, CT examinations identified skull destruction and calcification around the tumor in most patients, which was consistent with the results of many previous studies [12,13,14,17,37,38]. However, bone formation was only observed in the CT images of a few patients. Jelthi et al. reported a similar phenomenon in patients with limb and pelvis chondrosarcoma, with active bone absorption and actual bone remodeling [39], similar to cartilage formation.

Moreover, we analyzed the preoperative characteristics of parasellar chondrosarcoma and found that the tumors invaded related nerves in the cavernous sinus, inducing eye movement and dysfunction, diplopia, and ptosis, amongst other symptoms. This clinical feature was also reported in the relevant research, which mainly showed headache, decreased vision, strabismus, diplopia, and movement disorders [4,9,12,13,14,15,16,17,18,19,20,21]. Furthermore, CT and MRI examinations indicated that most patients had large tumors with upper clivus surrounding the sella. CTA showed that most tumors surrounded important arteries, including the internal carotid, middle cerebral, posterior cerebral, vertebral, and basilar arteries. It has also been reported that the cavernous carotid artery [14], the internal carotid artery [40], and the cavernous carotid artery [14] could be surrounded by parasellar chondrosarcoma. Generally, intracranial chondrosarcoma is difficult to resect completely and often requires adjuvant treatment for complete control [41]. Tumors with these preoperative characteristics greatly increase the difficulty of total resection of the parasellar chondrosarcoma.

In this study, three cases were treated using the endoscopic transnasal transsphenoidal approach, resulting in near-total resection. This surgical approach has certain advantages for removing tumors invading the inner-sellar area and midline and can remove tumors with minimal displacement of the surrounding tissues. However, this approach can result in residual tumors if the tumor deviates from the midline due to visual field defects. Although some tumors can be removed completely, this approach has been associated with a high recurrence rate [42].

Ten cases were treated using the transfrontotemporal approach, resulting in six total resections, one near-total resection, and three partial resections. Furthermore, two cases were treated using frontotemporal zygomatic arch transection, resulting in one partial resection and one near-total resection. The transfrontotemporal approach fully exposes the parasellar visual field, allowing direct observation of the tumor for removal. Therefore, protecting the surrounding blood vessels and nerves is easy. However, the zygomatic arch transection is more likely to expose tumors invading the middle-skull base.

We also analyzed risk factors associated with the degree of tumor resection. The resection degree was positively correlated with the preoperative KPS score. Furthermore, total resection did not correlate with important vessel wrapping or the maximum tumor diameter.

Pathologically, most tumors were grayish-white, hard, and tough during the operation. They also had a common blood supply, were honeycomb-like, and had clear boundaries. However, a few tumors were grayish-red and slightly soft. The focus usually grows in the bone by infiltration, which eventually breaks through the cortex and invades the surrounding soft tissue [1]. In this study, the postoperative pathological results identified WHO grades 1 and 2 with highly differentiated parasellar chondrosarcomas with significant cellular atypia and deeply stained binuclear cells in the nuclei. Additionally, mitosis was rarely observed. Furthermore, chondrosarcoma cells invaded the bone, causing bone destruction. The pathological results also determined that nine patients were negative for the IDH1 mutation, and the IDH1 mutation was undetectable in six patients. IDH1/2 mutations are associated with a low recurrence rate and high mortality [43]. Thus, evaluating these indicators may improve the prognostic accuracy for parasellar chondrosarcomas. Additionally, all patients were positive for S100, vimentin, and D2-40, and had low Ki-67 expression. However, chondrosarcoma does not express epithelial markers [44], which is a characteristic pathological result of chondrosarcoma.

All patients were followed-up, and no deaths were reported. Furthermore, for all patients, the postoperative KPS score was better than the preoperative KPS score in all patients. Only two patients relapsed or progressed within four to eight years after the surgery and underwent a reoperation. Generally, intracranial chondrosarcomas have a good long-term prognosis [45]. Our correlation analysis indicated that patients who underwent total chondrosarcoma resection were less likely to relapse than those who underwent partial resection; they also had longer progression-free survival time. A similar outcome was also reported in a skull base chondrosarcoma study [46]. In our study, the postoperative KPS score, maximum tumor diameter, WHO grade, important macrovascular surrounded by tumor, and age did not correlate with tumor recurrence. Yet, the postoperative KPS score was positively correlated with the preoperative KPS score and tumor resection degree and negatively correlated with the maximum tumor diameter and age. Additionally, improved KPS scores positively correlated with the tumor resection degree but not with the maximum tumor diameter, WHO grade, age, or the important vascular surrounded by tumor. Nevertheless, parasellar chondrosarcoma is rare, and more cases should be analyzed to confirm these correlations.

Although total tumor resection is important for treating parasellar chondrosarcoma, adjuvant therapy is sometimes required. In this study, three patients received 15 rounds of radiotherapy six months after the operation every other day for one month, one patient received four rounds of gamma knife radiotherapy, and one received cellular immunotherapy. The other patients did not require radiotherapy or chemotherapy. Unfortunately, chondrosarcoma is not sensitive to chemotherapy, and so far, no drug treatment has been approved. Nonetheless, several studies have reported a better prognosis in patients after maximal tumor resection, postoperative radiotherapy, and proton therapy [47,48,49].

Finally, most patients experienced postoperative complications, including tumor recurrence, cerebral infarction, epidural effusion, mastoiditis, arachnoid cysts, and atherosclerotic diseases that may result from radiation-induced protease expression [50]. Therefore, long-term postoperative follow-up is a good management strategy [47].

## 5. Conclusions

Parasellar chondrosarcomas are extremely rare. Patients with WHO grade 1 or 2 parasellar chondrosarcomas often have neuro-ophthalmological symptoms. In addition, the complete resection of parasellar chondrosarcomas is challenging. Our data further suggest that surgical resection can be used to effectively treat the disease. Therefore, as surgical technologies and lighting systems progress, and neuroendoscopy and craniotomy tools and techniques improve, so can the prognosis of these patients. Furthermore, we found that total tumor resection was more complicated if the patient had a low preoperative KPS score or if the tumor was large. However, a higher resection degree was associated with a greater increase in the KPS score after surgery, emphasizing the importance of resecting as much tissue as possible. Moreover, based on the degree of tumor resection, adjuvant radiotherapy and proton therapy may be suitable to reduce tumor recurrence, while immunotherapy is another viable postoperative adjuvant therapy option. In addition, young patients with a high KPS score and small tumors before surgery showed a better prognosis after total resection. In brief, KPS score, age, maximum tumor diameter, and resection degree are important prognostic factors in our study and may be confirmed in future research. Additionally, long-term follow-up is a feasible management strategy to detect tumor relapse.

## Figures and Tables

**Table 1:** Summary of preoperative clinical and radiological features in 15 patients.

Characteristic	Value
Sex	
Male	7
Female	8
Age group (years)	
Children (≤14)	1
Adolescents and young adults (15–39)	8
Adults (40–64)	5
Elderly (≥65)	1
Clinical manifestation	
Impaired eye function and movement	12
Dizzy and encephalagia	8
Tumor location	
Parasellar	13
Parasellar and intrasellar invasion	1
Parasellar and clivus eroded	1
Important vessels	
Not involved	5
Internal carotid artery	4
Middle cerebral artery	1
Internal carotid artery and middle cerebral artery	1
Internal carotid artery and posterior cerebral artery	1
Internal carotid artery, middle cerebral artery, and posterior cerebral artery	2
Internal carotid artery, vertebral artery, and basilar artery	1
Tumor size	
Maximum diameter of tumor (mm)	40 ± 13
Bone changes	
Bone destruction	7
Bone osteogenesis	2
Calcification	
Yes/No	15/0
Important vascular invasion	
Yes/No	10/5

**Table 2:** Surgical information of 15 patients with parasellar chondrosarcoma.

Case	Operative Approach	Excision Degree	Adjuvant Therapy	WHO Grade	Degree of Differentiation	*IDH1*	Postoperative Complications	Preoperative KPS	Postoperative KPS
1	Frontotemporal zygomatic arch approach	Partial excision	None	1	Highly differentiated	Negative	Recrudescence	60	65
2	Frontotemporal approach	Total excision	GKS	1	Highly differentiated	Negative	Epidural effusion, Mastoiditis	50	60
3	Frontotemporal approach	Total excision	None	1	Highly differentiated	Not measured	None	85	95
4	Frontotemporal zygomatic arch approach	Subtotal resection	None	1	Highly differentiated	Not measured	Epidural effusion, Mastoiditis	50	70
5	Frontotemporal approach	Total excision	None	1	Highly differentiated	Not measured	None	80	85
6	Neuroendoscope (EEA)	Subtotal resection	None	1	Highly differentiated	Negative	None	50	65
7	Frontotemporal approach	Subtotal resection	None	1	Highly differentiated	Negative	Arachnoid cyst	90	100
8	Neuroendoscope (EEA)	Subtotal resection	None	2	Highly differentiated	Negative	Mastoiditis	85	90
9	Frontotemporal approach	Total excision	None	1	Highly differentiated	Negative	Epidural hemorrhage	95	100
10	Frontotemporal approach	Total excision	None	2	Highly differentiated	Negative	Cerebral infarction, Epidural hematocele	80	95
11	Frontotemporal approach	Partial excision	RT	1	Highly differentiated	Not measured	Cerebral infarction	60	65
12	Frontotemporal approach	Total excision	RT	2	Highly differentiated	Negative	None	75	85
13	Neuroendoscope (EEA)	Subtotal resection	None	1	Highly differentiated	Negative	Mastoiditis	65	70
14	Frontotemporal approach	Partial excision	None	1	Highly differentiated	Not measured	Cerebral infarction	60	65
15	Frontotemporal approach	Partial excision	RT	1	Highly differentiated	Not measured	Subarachnoid hemorrhage	60	65

WHO grade: World Health Organization grade; *IDH1*: Isocitrate Dehydrogenase 1; GKS: gamma knife surgery; RT: Radiotherapy; KPS: Karnofsky Performance Scale.

**Table 3:** The risk factors correlated with resection degree were determined by Spearman correlation analysis.

Characteristic	R Value	*p* Value
Total excision		
Preoperative KPS	0.571	0.026
Important vascular surrounded by tumor	−0.107	0.705
Tumor maximum diameter	−0.070	0.803
Preoperative KPS		
Tumor maximum diameter	−0.745	0.001
Ages	−0.435	0.105
Important vascular surrounded by tumor	−0.382	0.160
WHO grade	0.313	0.256

Preoperative KPS: preoperative Karnofsky Performance Score; WHO grade: World Health Organization grade.

**Table 4:** Spearman correlation analysis of prognosis and KPS related factors.

Characteristic	R Value	*p* Value
Recurrence		
Total excision	−0.650	0.009
Postoperative KPS	−0.371	0.173
Maximum meridian of tumor	−0.274	0.322
WHO grade	−0.196	0.484
Important vascular surrounded by tumor	−0.139	0.622
Ages	0.136	0.628
Postoperative KPS		
Preoperative KPS	0.903	<0.001
Maximum meridian of tumor	−0.609	0.016
Total excision	0.571	0.026
Ages	−0.520	0.047
WHO grade	0.355	0.194
Important vascular surrounded by tumor	−0.335	0.223
KPS growth		
Total excision	0.536	0.039
Maximum meridian of tumor	0.355	0.194
WHO grade	0.191	0.496
Ages	−0.082	0.771
Important vascular surrounded by tumor	−0.018	0.949

Postoperative KPS: Postoperative Karnofsky Performance Score; WHO grade: World Health Organization grade; KPS growth: Karnofsky Performance Score growth.

## Data Availability

The data presented in this study are available in [“results” part and Supplementary Materials].

## Supplementary Materials

The following supporting information can be downloaded at: https://www.mdpi.com/article/10.3390/brainsci12101353/s1, Supplementary Table S1 Preoperative condition of 15 patients with parasellar chondrosarcoma.
