# Supplementary material for: Clinical Characteristics and Prognostic Risk Factors of Parasellar Chondrosarcoma
Source: Brain Sci. 2022 Oct 6;12(10):1353. doi: 10.3390/brainsci12101353 (PMC9599124; doi:10.3390/brainsci12101353)
Supplement: Supplementary file 1 [file brainsci-12-01353-s001.zip › brainsci-1911707-supplementary.pdf]

Online supplements

Supplementary Table S1 Preoperative condition of 15 patients with parasellar chondrosarcoma

Case	Age（yr）/Gender	Clinical manifestation	Physical examination	Tumor location	Tumor size(mm)	Bone destruction/ osteogenesis	CT	MRI	MRI Flair	MRI enhancement	Arterial encirclement	Invasion of related nerves in cavernous sinus
1	45/F	Visual double shadow 7 months, aggravated with dizziness 2 months	Abduction limitation of left eye	Left parasellar	28*36*22	Destruction	Lumpy high-low hybrid density shadow	Long T1 long T2	Uneven slightly higher signal	Lumpy uneven obvious enhancement shadow	Left internal carotid artery	Yes
2	49/F	Intracranial space occupying lesions were found for 2 years	Normal	Right parasellar and right CPA area	48*42*64	Destruction	Small enhanced shadow	Long T1 long T2	Slightly higher signal shadow	Quasi circular obvious enhancement shadow	Right carotid artery, middle cerebral artery	No
3	28/M	Binocular right diplopia 1 month	Right eye abduction slightly limited, diplopia occurs when both eyes look right	Right parasellar	32*25*25	Destruction	Lumpy uneven slightly low density shadow	Equal length T1 long T2	Slightly higher signal shadow	Significantly uneven honeycomb reinforceme nt	Not involved	Yes
4	30/F	Left eye pain 10 days	Normal	Left Petrocliv al area, left parasellar	62*50*37	Destruction	Large density shadow	Uneven long T1, high-low mixed T2	Uneven slightly low signal shadow	Lobulated lumpy uneven obvious enhanced shadow	Left internal carotid artery	No
5	26/F	Dizziness, double vision with ptosis of left eye 2 months	Ptosis of left eye	Left parasellar and petrous apex	35*20*20	Destruction	Small uneven slightly high-densi ty shadow	Slightly longer T1 slightly longer T2	Slightly higher signal	Obvious uneven enhancement , meningeal tail sign	Not involved	Yes
6	31/M	Visual double shadow and abduction disorder in the left eye 6 years	Abduction disorder of left eye, diplopia of left, upper and lower eyes	Left parasellar , clivus	48*22*32	No	Irregular lumpy high-low hybrid density shadow	Uneven long T1 long T2	Slightly higher signal	Large uneven enhancement shadow	Left internal carotid artery, vertebral artery and basilar artery	Yes
7	22/M	Headache for 7 days, double vision 5 days	Double eye right vision double shadow, right eye abduction Limited	Right petrous apex and parasellar	21*24*19	No	Small lumpy high-low hybrid density shadow	Uneven length T1 long T2	High signal shadow	Small uneven obvious enhancement shadow	Not involved	Yes
8	43/F	5 days after tumor discovery	Numbness in the right face, decreased vision in the right eye, limited abduction, decreased hearing in the right ear	Right parasellar	29*34*35	Osteogenesis	Large low density shadow	Uneven length T1 long T2	Slightly higher signal	Lumpy uneven enhancement shadow	Bilateral internal carotid arteries	Yes
9	14/M	Headache 2 months, eye movement disorder 1 month	The left eye fixed, with limited movement, dullness above the left eye fissure, allergy below the eye fissure and above the mouth and lip	Left parasellar	29*33*34	Osteogenesis	Lumpy low density shadow	Long T1 long T2	Uneven slightly low signal	Lumpy uneven enhancement shadow	Left internal carotid artery	Yes
10	34/F	Intermittent	Decreased visual	Right	36*33*26	No	Lumpy	Long T1	Uneven	Lumpy	Right internal	Yes

		headache 15 months, right eye vision decreased 5 months, left eye vision decreased 2 months	acuity in both eyes, romberg's sign positive	parasellar			low enhancement shadow	long T2	low density shadow	uneven light to moderate enhanced shadow	carotid artery, middle cerebral artery and posterior cerebral artery	
11	27/M	Hemiplegia of right limbs with speech impairment 5 months, visual acuity of left eye decreased 2 months	The direct light reflex of the left eye disappeared, the indirect light reflex of the right eye disappeared, the muscle strength of the right upper limb was grade 3, the distal end was grade 1, and the muscle strength of the right lower limb was grade 4	Left parasellar	56*43*43	No	Lumpy high-low hybrid density shadow	Uneven length T1 long T2	Uneven low signal	Uneven strengthening	Infarction of left middle cerebral artery and basal ganglia	Yes
12	46/M	Right eye pain half a month	Right eyeball slightly protrudes	Right clivus, parasellar and intrasellar	36*35*35	No	Irregular lumpy uneven slightly low density shadow	Uneven length T1 long T2	Uneven low signal	Lumpy uneven obvious enhancement shadow	Not involved	No
13	69/F	Intermittent dizziness 21 years, right facial numbness 8 months, right eye strabismus 6 months	Strabismus of right eye	Right parasellar ,petrous apex	35*24*32	Destruction	Low intensity shadow	Uneven length T1 long T2	Uneven high signal	Lumpy uneven enhancement shadow	Right internal carotid artery, posterior cerebral artery, left middle cerebral artery is slightly thin, and bilateral internal carotid arteries are sclerotic	Yes
14	33/M	Left temporal distension and pain with binocular vision loss 12 days	Poor abduction of both eyes	Left parasellar and suprasellar	16*22*20	Destruction	Lumpy high-low mixed density shadow and calcification shadow, mainly calcification	Long T1 slightly long T2	Slightly higher signal	Uneven obvious strengthening	Not involved	Yes
15	52/F	Right side headache 1.5 years, right side eyelid ptosis with visual double shadow 1 month	Right eyeball adduction, insufficient up and down vision, blurred vision and diplopia in the right eye	Right clivus and parasellar	46*23*33	Destruction	Uneven low intensity shadow	Uneven length T1 long T2	Slightly higher signal	Uneven intensifying shadow	Right internal carotid artery, posterior cerebral artery	Yes

(yr: year; M: Male; F: Female; CT: Computed Tomography; MRI: Magnetic Resonance Imaging)
